# Dr. Clarence B. LeVan

**Published:** 1903-09

**Authors:** 


Dr. Clarence B. LeVan, of Buffalo, died at his residence after a prolonged illness, July 26, 1903, aged 39 years. He was a graduate of Niagara University Medical College, and after about ten years' practice his health began to fail, causing him to abandon his professional work three years ago. He is survived by a widow and two children.

